# Early life growth and developmental trajectory in children with biliary atresia undergoing primary liver transplantation

**DOI:** 10.3389/fped.2023.1198360

**Published:** 2023-06-12

**Authors:** Heping Fang, Zehao Li, Ruoling Xian, Yu Yin, Juan Wang, Hongling Guo, Xiaoke Dai, Mingman Zhang, Yan Hu, Yingcun Li

**Affiliations:** ^1^ Department of Child Health Care, Children’s Hospital of Chongqing Medical University, Chongqing Key Laboratory of Pediatrics, Ministry of Education Key Laboratory of Child Development and Disorders, National Clinical Research Center for Child Health and Disorders, Chongqing, China; ^2^ Department of Hepatobiliary Surgery, Children's Hospital of Chongqing Medical University, Chongqing Key Laboratory of Pediatrics, Ministry of Education Key Laboratory of Child Development and Disorders, National Clinical Research Center for Child Health and Disorders, Chongqing, China

**Keywords:** biliary atresia (BA), primary liver transplantation (pLT), growth and development, early life, children

## Abstract

**Objective:**

To clarify the early growth and developmental characteristics of children with biliary atresia (BA) undergoing primary liver transplantation (pLT).

**Methods:**

A prospective cohort study, which specifically focused on BA-pLT children, was conducted after the diagnosis of BA by following the children at the time of pLT and 1, 3, 5, 7 months and 1 year after pLT for growth and developmental monitoring. The growth parameters were calculated according to the WHO standard, and the developmental status was assessed using Denver Developmental Screening Tests.

**Results:**

A total of 48 BA children who received pLT at the age of 5.00 ± 0.94 months were analyzed. The weight-for-age *Z-*value (*Z*_W_) and length-for-age *Z*-value (*Z*_L_) were higher than the head circumference-for-age *Z-*value (*Z*_HC_) at pLT (*P *= 0.002 and 0.02), but they were all lower than the WHO growth standard (*Z* = 0) (*P *< 0.001). The *Z*_W_ and *Z*_HC_ decreased first and then returned to the population level at 1 year after pLT, while the *Z*_L_ only returned to the preoperative status and was lower than the *Z*_W_ and *Z*_HC_ (*P *< 0.001). Developmental screening showed that 35% (17/48) of the children were defined as suspicious and 15% (7/48) were abnormal at 1–4 months after pLT, the most likely time to be suspected of developmental delay. At 1 year after pLT, gross motor skill delay still existed (12/45, 27%), and language skill delay began to appear (4/45, 9%).

**Conclusions:**

BA-pLT children suffer from growth and developmental problems. Low *Z*_HC_ is the main growth problem before pLT, while low *Z*_L_ is the problem after pLT. Developmental delays are significant after pLT, especially in motor and language skills. The current study suggested that further studies are warranted to clarify the long-term growth and developmental outcomes of BA-pLT children, to compare them with children undergoing the Kasai procedure and to explore their influencing factors and possible mechanisms.

## Introduction

Biliary atresia (BA) is an infantile cholestatic liver disease caused by congenital structural abnormalities and/or progressive fibrosis of the biliary tract, which is the most common cause of pediatric liver transplantation (LT). The incidence of BA in China (1/9,200) (1) is higher than that in Western countries (1/18,000–1/12,000) (2). It can be estimated that the number of children with BA will increase by more than 1,000 every year (3), which results in huge expenditures on social and medical resources (refer to the data from Japan) (4).

Currently, surgery is essential to treat BA; otherwise, patients will die before 2 years of age. The main surgical methods include Kasai portoenterostomy (KPE) and LT, of which KPE is a palliative operation and LT is a curative operation. It was originally suggested that early KPE could improve the prognosis, delaying or even avoiding LT (5). Thus, LT was considered a salvage surgery and was provided when KPE failed, forming a sequential treatment procedure of “KPE-LT”. However, the efficacy of KPE has been controversial for a long time, and the survival rates of 1,340 BA patients with native liver after KPE were 41%, 35%, 26% and 22% at 5, 10, 20 and 30 years in France, respectively (6). Therefore, it has been suggested that bypassing KPE and carrying out primary LT (pLT) may achieve a better prognosis (7, 8), which has led to the increasing cases of BA children receiving pLT (BA-pLT) worldwide in the past 30 years (0.8%–15%) (9–11). In addition, given the liver source shortage in some regions due to ethical and religious reasons, the proportion of BA-pLT children receiving living donor LT is increasing dramatically. These children need to be specifically studied, as they are considered to be the youngest children receiving LT.

Pediatric chronic liver diseases (CLDs) are often accompanied by nutritional disorders (12), which are also common in BA children and affect their prognosis (13). In addition, BA children will suffer from growth disorders, which begin in infancy and could persist until adolescence (14, 15). BA children will also suffer from developmental disorders even if they receive successful KPE (16, 17). However, the current data mainly came from children receiving KPE or KPE-LT, and it remains unclear whether their experience is suitable for BA-pLT children. Based on this, our study specifically focused on BA-pLT children, aiming to clarify their growth and developmental trajectories in early life and to provide evidence for further postoperative management.

## Methods

### Study design and population

This study was a prospective observational cohort study conducted in the Department of Child Health Care and the Department of Hepatobiliary Surgery, Children's Hospital of Chongqing Medical University and approved by the Medical Research Ethics Committee (No. 2019-214). This study followed the Declaration of Helsinki and Istanbul, and informed consent was obtained from the children's caregivers. In addition, all surgery programs in this study were processed, discussed and approved by the medicine specialist group in Chongqing, whose qualifications were certificated by the Chongqing Health Commission. The results are reported in accordance with the STROBE (cohort studies) statement.

**Figure 1:**
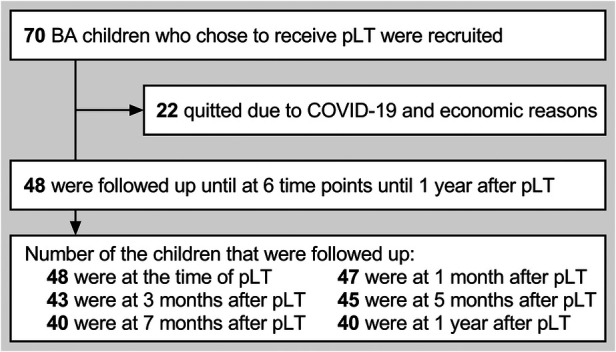
Follow-up plan and implementation of this study. BA, biliary atresia; pLT, primary liver transplantation.

From September 2019 to August 2021, we recruited 70 children with BA who were diagnosed by biliary exploration, and decided to receive pLT by parents after understanding the relevant information on KPE and pLT (all children received living donor LT due to the shortage of deceased organ donation) (18). Forty-eight children were followed up until 1 year after pLT (22 children quitted because of the COVID-19 epidemic and economic reasons, and some children failed to keep the follow-up plan for similar reasons). In summary, we evaluated the children within 5 days before pLT (defined as “at pLT”) and at 1 month, 3 months, 5 months, 7 months, and 1 year after pLT, and recorded the data about growth and developmental conditions (Figure 1). The main outcome of this study was the growth trajectory of BA children within 1 year after pLT, while their developmental status was the secondary outcome.

### Sample size estimation

The sample size was estimated based on the formula summarized by Serdar et al. (19) It was reported that the *Z*-value of the length of BA children within 2 years old who received LT was −1.8 ± 1.4 (20). Thus, we took this value as a reference when estimating the sample size, and the calculation provided that a minimum sample size of 30 children could estimate the two-sided 95% confidence interval with an allowable error of 0.5.

### Anthropometric and developmental measurements

Anthropometric (weight, length and head circumference) and developmental (Denver Developmental Screening Tests, DDST) measurements were conducted by trained pediatric nurses and interpreted by trained pediatricians. The weight-for-age *Z-*value (*Z*_W_), length-for-age *Z*-value (*Z*_L_) and head circumference-for-age *Z*-value (*Z*_HC_) were calculated by software according to the WHO standards (WHO Anthro) (21). Values of *Z*_W_, *Z*_L_ and *Z*_HC_ under −2 were defined as “abnormally low” in this study. DDST included tests on 4 developmental skills (gross motor, fine motor-adaptive, language, and personal-social skill) and was conducted only when the doctor suspected that the child had a delay through medical history inquiry (22). The conclusions were divided into “abnormal, suspicious and normal” according to the number of delayed items (Table 1) (23). In addition, skills with 2 or more delayed items were defined as “developmental delay” in this study.

**Table 1:** The criteria for the conclusions of DDST.

Conclusions	Definitions of the criteria
Delayed item	The items that the children can not complete are recorded as “failed items”, and the failed items are defined as ‘delayed items’ when the ages of the tested children exceed 90% of the allowable age range of the items.
Normal	Without the conditions above.
**Suspicious**	
Condition 1	One developmental skill has two or more delayed items.
Condition 2	One or more developmental skills have one delayed item with all items on the age line failed.
**Abnormal**	
Condition 1	Two or more developmental skills, each has two or more delayed items.
Condition 2	One developmental skill has two or more delayed items, and one or more developmental skills have one delayed item with all items on the age line failed.

DDST, Denver Developmental Screening Tests.

### Feeding guidance and oral nutritional supplementation

All children were given feeding guidance and oral nutritional supplementation at each follow-up time point by Child Health Care doctors (Child Health Care doctors provide children's routine feeding and nutritional guidance in China). The total energy intake was recommended to be 130%–150% of the reference energy requirement before pLT, and was recommended to be 120% of the reference energy requirement after pLT, which was in accordance with the recommendations of nutritional support for children with CLD and the reference dietary nutrient intake of Chinese residents (24, 25). However, data about the exact energy intake were not collected due to a lack of attention from caregivers (nutritional problems were commonly ignored by caregivers due to the lack of health education and compliance), and feeding guidance and oral nutritional supplements were applied according to the anthropometric and developmental data of the children instead of energy intake (26).

For the medication plan, all children received the same immunosuppressive treatment after pLT. Methylprednisolone was administered intravenously after surgery and was gradually reduced to oral dexamethasone tablets under blood concentration monitoring until 3 months after surgery. In addition, oral tacrolimus or cyclosporin was used at the next morning after surgery, and mycophenolate mofetil would be added if an acute rejection was suspected.

### Data processing and statistical analysis

Data analysis was performed by GraphPad Prism 9. Qualitative data are described as numbers (percentages), and quantitative data are described as the mean (standard deviation, SD) after normality testing. We plotted the growth trajectories using the mean ± SD, calculated the difference in *Z*-values (Δ*Z*-value) at adjacent time points and plotted the trajectories of the Δ*Z*-values at the average level. Repeated-measures ANOVA, paired *t* test and one sample *t* test were used to compare the differences among *Z*_W_, *Z*_L_ and *Z*_HC_, and the differences with the WHO population level (*Z* = 0). *P *< 0.05 was considered statistically significant.

Before analysis, the five pre-term children were evaluated separately, and their *Z*-values were corrected according to gestational age of 40 weeks. They were finally included in the formal analysis because their growth pattern was similar to that of full-term children (Supplementary Figure S1). Then, we evaluated the baseline characteristics of the 22 quit children and found no significant differences from the children who were followed up. In addition, a total of 8.7% (75/864) of the anthropometric data of the children followed up were missing. We evaluated the differences in data between the missing value group and the non-missing value group at the next follow-up time point and found no significant differences. Thus, the missing data were treated as missing completely at random and no assumptions were made.

## Results

### General characteristics

Table 2 shows the general characteristics and age at each follow-up time point of the 48 BA-pLT children. The results of the growth and developmental status were not different between males and females, as no significant differences at each follow-up time point were found. Further analysis showed that the *Z*_W_-value of the children at birth (−0.13 ± 0.86) was not significantly different from the WHO population level (*P *= 0.31).

**Table 2:** General characteristics in BA children (*n* = 48).

Characteristics	Results
**Sex, *n* (%)**	
Male	25 (52)
Female	23 (48)
*Z*_W_ at birth, mean (SD)	−0.13 ± 0.86
Father's height (cm), mean (SD)	170.86 ± 4.67
Mother's height (cm), mean (SD)	158.16 ± 4.36
Age at diagnosis (month), mean (SD)	2.62 ± 1.24
Age at pLT (month), mean (SD)	5.00 ± 0.94
**Age of follow up (month), mean (SD)**	
At pLT	4.82 ± 0.83
1 month after pLT	6.21 ± 1.00
3 months after pLT	8.08 ± 1.07
5 months after pLT	9.95 ± 1.04
7 months after pLT	12.23 ± 1.15
1 year after pLT	17.54 ± 1.24

Values are expressed as number (%) and mean (SD), decimal places are not use as sample size is less than 200. BA, biliary atresia; SD, standard deviation; pLT, primary liver transplantation; *Z*_W_, weight-for-age *Z* score.

### Growth trajectory of the BA-pLT children

**Figure 2:**
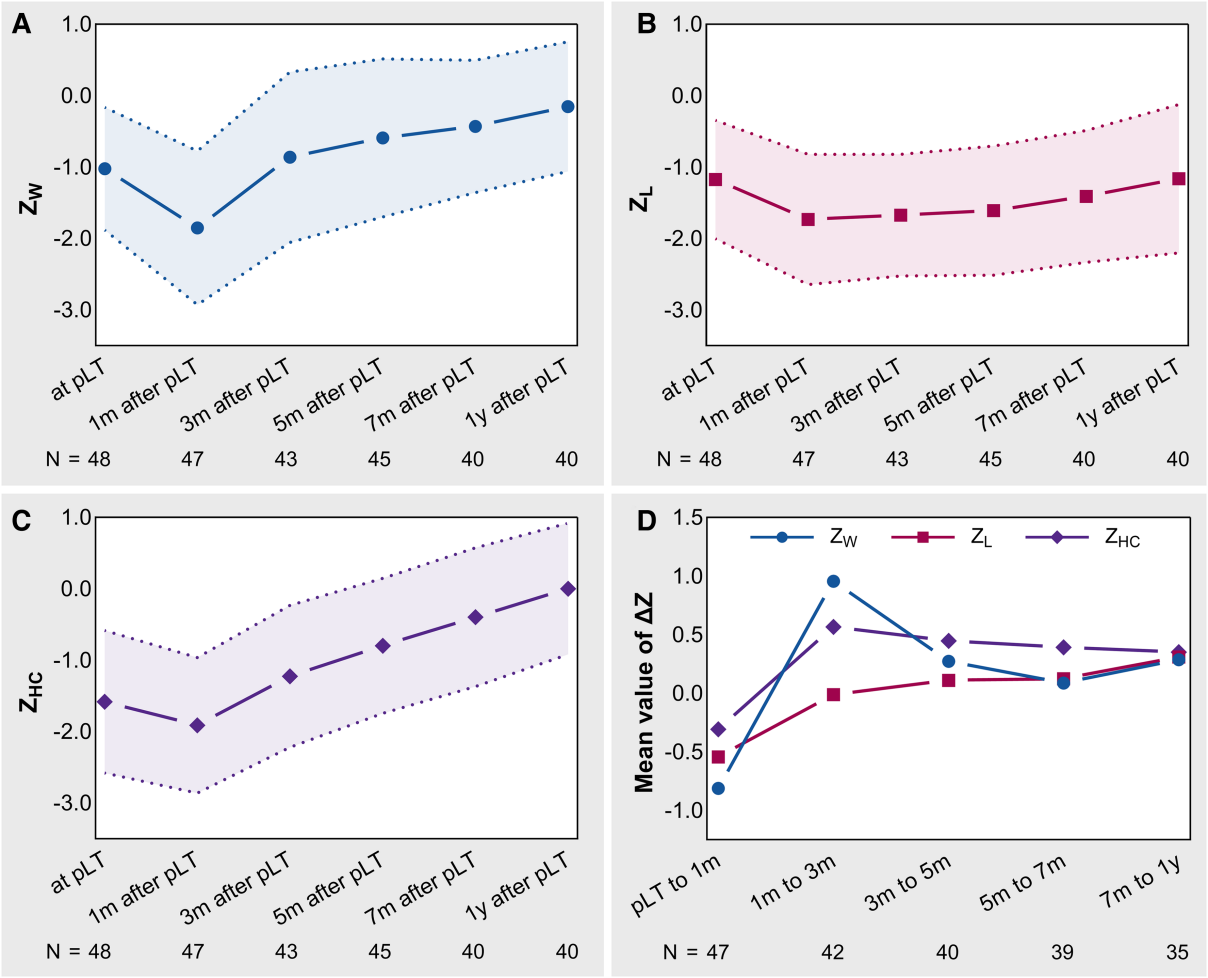
The trajectories of *Z*-values and Δ*Z*-values in BA-pLT children. (**A**) Growth trajectory of *Z*_W_; (**B**) Growth trajectory of *Z*_L_; (**C**) Growth trajectory of *Z*_HC_; (**D**) Growth trajectories of Δ*Z*-values. BA, biliary atresia; pLT, primary liver transplantation; *Z*_W_, weight-for-age *Z*-value; *Z*_L_, length-for-age *Z*-value; *Z*_HC_, head circumference-for-age *Z*-value.

Figure 2 shows the trajectories of the *Z*-values and Δ*Z*-values. *Z*_W_ and *Z*_HC_ showed a pattern of first descending and then rising, with the lowest point at 1 month after pLT (Figures 2A,C). However, *Z*_L_ reached a plateau at 1 month after pLT, declined and then recovered slowly (Figure 2B). In addition, *Z*_W_ and *Z*_HC_ had significantly accelerated growth (Δ*Z* > 0) after pLT, which gradually slowed down over time, especially *Z*_W_. In contrast, *Z*_L_ maintained slow but showed steady accelerated growth within 1 year after pLT (Figure 2D).

### Growth status at pLT and 1 year after pLT

**Figure 3:**
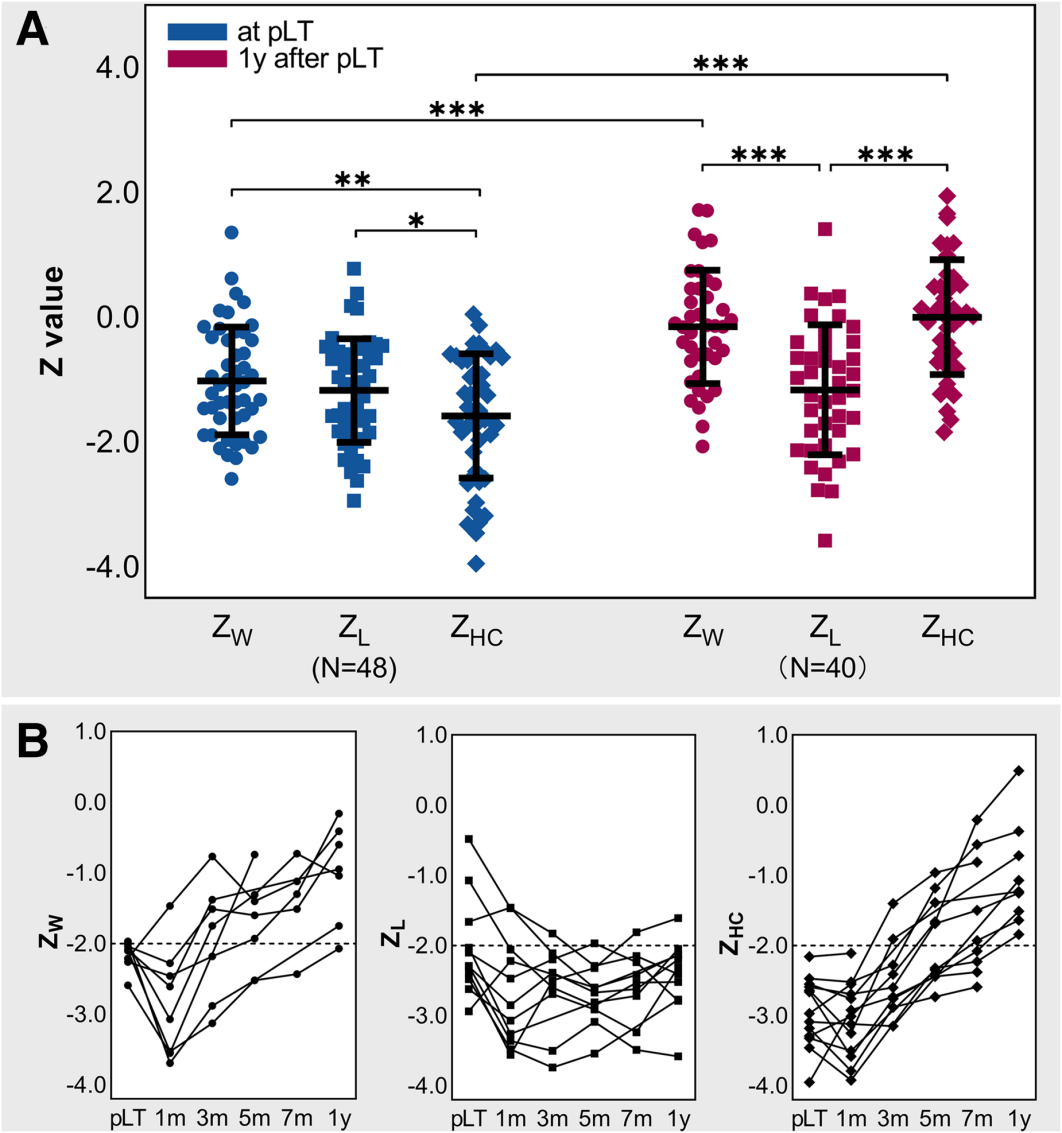
Growth status at pLT and 1 year after pLT. (**A**) *Z*-values at pLT and 1 year after pLT in BA children; repeated-measures ANOVA and paired *t* test; *P < 0.05, **P < 0.01, ***P < 0.001. (**B**) Trajectories of *Z*-values of BA-pLT children with *Z*-value lower than −2. BA, biliary atresia; pLT, primary liver transplantation; *Z*_W_, weight-for-age *Z*-value; *Z*_L_, length-for-age *Z*-value; *Z*_HC_, head circumference-for-age *Z*-value.

Figure 3A shows the *Z-*values at pLT and 1 year after pLT. Paired analysis showed that *Z*_W_ (−1.09 ± 0.86) and *Z*_L_ (−1.09 ± 0.83) were significantly higher than *Z*_HC_ (−1.52 ± 1.00) at pLT (*P *= 0.002 and 0.02), and the three of them were all significantly lower than the WHO population level (*P *< 0.001). In addition, although *Z*_W_ (−0.15 ± 0.91) and *Z*_HC_ (0.02 ± 0.92) improved significantly within 1 year after pLT (*P *< 0.001), *Z*_L_ (−1.12 ± 1.04) did not (*P *= 0.83) and was even significantly lower than *Z*_W_ and *Z*_HC_ (*P *< 0.001). Moreover, *Z*_W_ and *Z*_HC_ recovered to the WHO population level at 1 year after pLT (*P *= 0.29, *P *= 0.99), while *Z*_L_ was still significantly lower than the WHO population level (*P *< 0.001).

Figure 3B shows the growth trajectories of the children whose *Z-*values were defined as abnormally low at pLT and/or 1 year after pLT (note that some data were missing). At pLT, 15% (7/48) of children had abnormally low *Z*_W_, 17% (8/48) had abnormally low *Z*_L_, and 27% (13/48) had abnormally low *Z*_HC_. At 1 year after pLT, 3% (1/40) of children had abnormally low *Z*_W_, 25% (10/40) had abnormally low *Z*_L_, and none had abnormally low *Z*_HC_. Further analysis showed that 7 of the 8 children with abnormally low *Z*_L_ at pLT still had abnormally low *Z*_L_ at 1 year after pLT, and 8% (3/40) of the children with normal *Z*_L_ at pLT (*Z* = −1.66, −0.48, −1.07) had abnormally low *Z*_L_ (*Z* = −2.14, −2.41, −2.52) but normal *Z*_W_ (*Z* = −0.70, −1.17, −1.34) and *Z*_HC_ (*Z* = 0.52, −0.41, −0.07) at 1 year after pLT. Notably, the father's height and mother's height of those who had abnormally low *Z*_L_ at 1 year after pLT were not significantly different from others (*P *= 0.25 and 0.57).

### Developmental trajectory of the BA-pLT children

Table 3 shows the developmental status and DDST results of the BA-pLT children. One to four months after pLT was the time when children were most likely to be suspected of developmental delay. At that time, 35% (17/48) of the children were defined as suspicious, and 15% (7/48) were defined as abnormal. Further analysis showed that 15% (7/48) of the BA children had developmental delay of gross motor or fine motor-adaptive skills at pLT. In addition, the developmental delay of fine motor-adaptive skills gradually disappeared at 1 year after pLT, but the delay of gross motor skills still existed (12/45, 27%), and a delay of language skills began to appear (4/45, 9%).

**Table 3:** The results of Denver Developmental Screening Tests.

Results	At pLT (*N* = 48)	1–4 m after pLT (*N* = 48)	4–8 m after pLT (*N* = 47)	8 m-1 year after pLT (*N* = 45)
Negative history, *n* (%)	30 (63)	14 (29)	19 (40)	20 (44)
Positive history, *n* (%)	18 (38)	34 (71)	28 (60)	25 (56)
Normal	8 (17)	10 (21)	9 (19)	10 (22)
Suspicious	6 (13)	17 (35)	13 (28)	9 (20)
Abnormal	4 (8)	7 (15)	6 (13)	6 (13)
**Developmental delay, *n* (%)**				
Gross motor	4 (8)	15 (31)	12 (26)	12 (27)
Fine motor-adaptive	4 (8)	5 (10)	0 (0)	0 (0)
Language	0 (0)	1 (2)	3 (6)	4 (9)
Personal-social skill	0 (0)	0 (0)	0 (0)	0 (0)

Values are expressed as number (%), decimal places are not use as sample size is less than 200. pLT, primary liver transplantation.

## Discussion

This study specifically focused on the early life growth and developmental characteristics of BA-pLT children, who are considered to be the youngest group of BA children undergoing LT. We found that the *Z*_W_ and *Z*_HC_ of the children decreased to their lowest level at 1 month after pLT and then gradually returned to normal within 1 year after pLT. However, *Z*_L_ did not show significant improvement compared to its baseline level and remained lower than normal even at 1 year after pLT. Furthermore, we found that BA children may experience significant developmental delays after pLT, particularly in motor and language skills.

Our study revealed that *Z*_W_ dropped to the lowest point at 1 month after pLT, which may be attributed to the complexity of LT surgery and the need for multiple postoperative treatments. In addition, we observed a significant improvement in *Z*_W_ within 1 year after pLT, which is in line with previous research on pediatric LT patients with end-stage liver diseases (27, 28). The short-term improvement in *Z*_W_ after surgery may not only be attributed to the removal of multiple postoperative treatments, but also to the withdrawal of glucocorticoids. However, this study was unable to confirm the correlation between the two due to the lack of information on the duration of glucocorticoid administration in all individuals after surgery.

Previous studies have shown that the catch-up growth of LT children (3.89 ± 0.28 years old) after surgery can be categorized into three stages: acceleration (occurring within the first year after surgery, but not reaching normal height level), plateau (occurring between 1 and 10 years after surgery), and deceleration (occurring between 10 and 15 years old) (20, 29). The acceleration stage was more pronounced in younger children (especially under 2 years old) (27–29), which indicated that providing LT to BA children at a younger age could be more effective in promoting the catch-up growth of *Z*_L_ after surgery. However, our study did not obtain the expected results. Despite exhibiting slow accelerated growth within 1 year after surgery, *Z*_L_ only returned to its preoperative level. Additionally, three children who had normal *Z*_L_ at pLT experienced a drop to abnormal levels (*Z* < −2) at 1 year after pLT, although their *Z*_W_ and *Z*_HC_ remained normal. Insufficient energy intake may be the reason for the observed issue, as pLT (5.00 ± 0.94 months old) was at an age where the energy density of liquid food becomes insufficient and needs to be supplemented (typically between 4 and 6 months old). Encouraging parents to appropriately advance the time for introducing complementary foods and conducting nutrient testing for early intervention could potentially help address this issue. In this aspect, although more comprehensive research is needed, we have condensed our practical experience into a simplified flowchart (Supplementary Figure S2). Given that energy intake and utilization play a more significant role than genetic factors in the growth of *Z*_L_ in BA children, especially those under the age of 2 (30), we cannot provide further interpretations as we did not collect their energy intake data. In addition, our study showed that the *Z*_L_-values that were below −2 at pLT remained below −2 even at 1 year after pLT, implying that the status of *Z*_L_ at pLT may be linked to catch-up growth after surgery, as reported by Mohammad et al. (31) In summary, although the height of BA children after the KPE-LT procedure may still be lower than the WHO population level (29), it is unclear whether BA-pLT children can achieve better growth outcomes than KPE-LT children. Therefore, further studies are warranted to determine this.

Currently, few studies have been conducted on the growth of *Z*_HC_ in BA children. Sokol et al. (32) showed a decrease (−0.46 ± 1.24) in the *Z*_HC_ of children under 2 years old with CLD. Our study, on the other hand, discovered that the mean level of *Z*_HC_ in BA children at pLT was −1.52, with 27% of them having abnormally low *Z*_HC_, which was significantly worse than *Z*_W_ and *Z*_L_ at the same time, indicating that the *Z*_HC_ of BA-pLT children was inferior to that of CLD children, especially when pLT was not performed. Therefore, the growth of *Z*_HC_ in BA-pLT children may be a significant problem that has been overlooked in the past. It is worth noting that despite both *Z*_HC_ and *Z*_L_ being involved in bone growth, their growth trajectories after pLT were not consistent. While *Z*_HC_ was significantly lower than *Z*_L_ at the time of pLT, it was significantly higher than *Z*_L_ at 1 year after surgery. Given that the growth of *Z*_L_ is related to the growth plate and is regulated by the growth hormone/insulin-like growth factor-1 axis (33), it could be affected by BA (34, 35). However, no evidence has suggested that *Z*_HC_ is also regulated by the growth hormone/insulin-like growth factor-1 axis. Instead, *Z*_HC_ was reported to be related to neurodevelopment and was affected by cholestasis (36). Therefore, we speculate that BA inhibits neurodevelopment by causing cholestasis, leading to the inhibition of *Z*_HC_ at the time of pLT and its acceleration after the removal of cholestasis, which is worthy of further study.

According to the DDST, a significant percentage of BA-pLT children (20%–50%) were found to be at risk of developmental delay, either suspicious or abnormal. Notably, some of the children were not tested by DDST based on the doctors' judgment and were classified as non-developmental delay, which may result in an underestimation of the actual risk. Our results are consistent with previous reports that BA children experience impaired motor and language skills at LT (37). This could be due to a decrease in intestinal fat absorption, resulting in a lack of long-chain polyunsaturated fatty acids that can cause developmental delay within 8–12 weeks (25). In addition, Talcott et al. reported that the long-term cognitive abilities of children with CLD were related to the duration of their liver disease rather than its severity (36). Thus, early intervention to relieve biliary obstruction and restore liver function may help mitigate developmental delays in BA children. In this regard, it should be emphasized that although the age of LT in BA-pLT children (5.00 ± 0.94 months old) was younger than that in KPE-LT children [the mean difference was 12.90 months according to Liu et al. (38)], the time of cholestasis was longer in our study (5.00 months) than in the study by Chen el al. (73.4 days) (39). However, it is still unclear whether the risk of developmental delay in BA-pLT children is different from that in KPE-LT children, suggesting that more attention should be given to the neurodevelopmental status of BA-pLT children.

### Limitations

Although our study described intriguing findings, it is important to acknowledge its limitations. First, the study was conducted at a single center and had a short-term follow-up, thus necessitating further research in the future. Second, despite focusing on the growth and development of the children, the absence of energy intake data made it difficult to fully interpret the results. Last, it is worth noting that DDST is primarily a screening test and that its results require validation from more accurate tests.

## Conclusions

In conclusion, our study indicates that BA-pLT children experience distinct growth and developmental issues compared to traditional LT children. While BA-pLT children are able to make significant improvements in *Z*_W_ and *Z*_HC_ within the first year after pLT, they do not experience the same level of improvement in *Z*_L_. Low *Z*_HC_ is the primary growth issue before pLT, whereas low *Z*_L_ becomes the main concern after surgery. In addition, BA-pLT children are at a high risk of developmental delays, especially in motor skills, and language developmental delays may occur gradually after pLT. Further studies are warranted to clarify the long-term growth and developmental outcomes of BA-pLT children, to compare them with those of KPE children and to explore their influencing factors and possible mechanisms.

## Data Availability

The original contributions presented in the study are included in the article/**Supplementary Material**, further inquiries can be directed to the corresponding author.

## Supplementary material

Supplementary Figure S2.

Flowchart of introducing complementary foods for BA-pLT children. BA, biliary atresia; pLT, primary liver transplantation.
