# Supplementary figures and images for: Early life growth and developmental trajectory in children with biliary atresia undergoing primary liver transplantation
Source: Front Pediatr. 2023 Jun 12;11:1198360. doi: 10.3389/fped.2023.1198360 (PMC10291188; doi:10.3389/fped.2023.1198360)

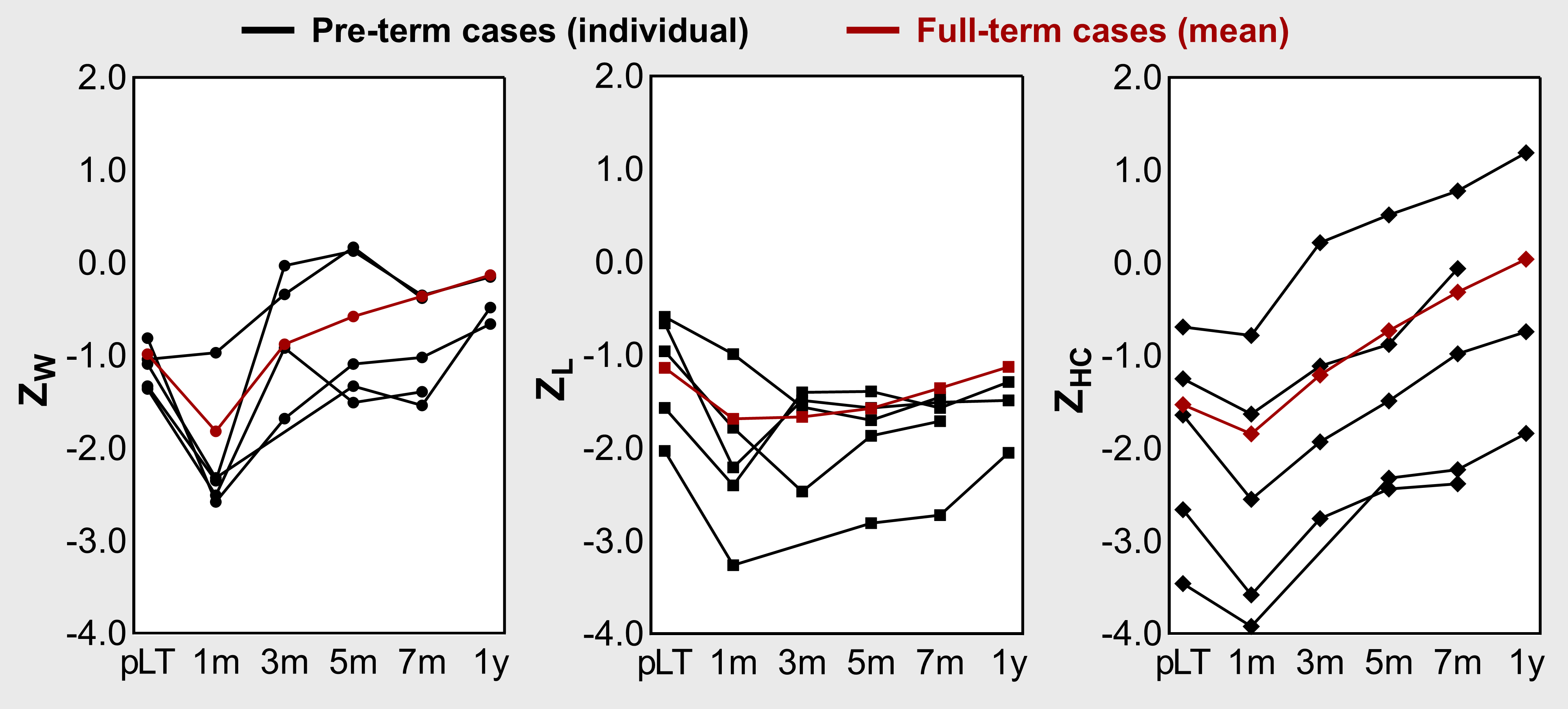

Supplement: Supplementary file 1 [file Image1.tif]

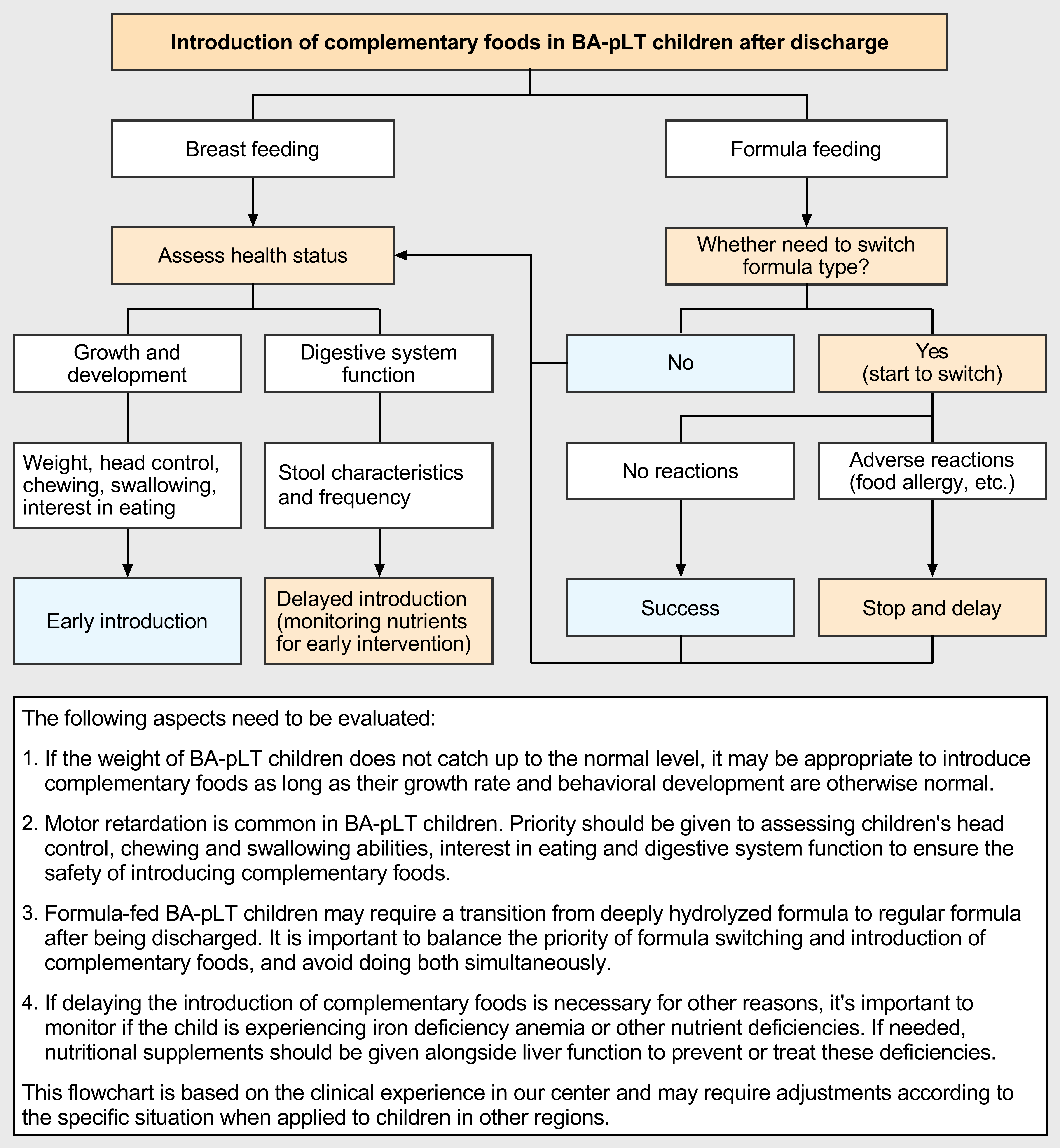

Supplement: Supplementary file 2 [file Image2.tif]
